# Perceived Cognitive Decline in Multiple Sclerosis Impacts Quality of Life Independently of Depression

**DOI:** 10.1155/2014/128751

**Published:** 2014-09-01

**Authors:** Lampros Samartzis, Efthymia Gavala, Yiannis Zoukos, Achilleas Aspiotis, Thomas Thomaides

**Affiliations:** ^1^Department of Neurology, General Hospital of the Greek Red Cross “Korgialeneio-Benakeio”, Athens, Greece; ^2^Department of Psychiatry, Athalassa Mental Health Hospital, Nicosia, Cyprus; ^3^St. George's University of London Medical School, University of Nicosia, Nicosia, Cyprus; ^4^Department of Neurology, St. Bartholomew's Royal London and Broomfield Hospitals, London, UK

## Abstract

*Background/Aim*. The aim of this study is to examine the effects of perceived cognitive dysfunction and of depression, on self-reported QoL, in a Greek population sample of MS patients.* Methods*. One hundred outpatients diagnosed with MS completed the Short-Form-36 Health Survey (SF-36), as well as the Perceived Deficits Questionnaire (PDQ) and the Depression subscale of the Mental Health Inventory (MHI), as part of a clinical evaluation which included the Expanded Disability Status Scale (EDSS) estimation. Multiple linear regression was conducted to determine the best linear combination of age, gender, education, EDSS, depression, attention/concentration, retrospective memory, prospective memory, and planning/organization, for predicting QoL scores.* Results*. In the multivariate regression analysis models, EDSS (*P* < 0.05), depression (*P* < 0.001), perceived planning/organization (*P* < 0.05), and perceived retrospective memory dysfunction (*P* < 0.05) independently predict quality of life scores. Age, sex, education level, and perceived attention/concentration dysfunction, as well as perceived prospective memory dysfunction, do not independently predict quality of life scores.* Conclusions*. Perceived planning/organization impairment and perceived retrospective memory impairment in MS patients predict QoL independently of the severity of disease and the severity of depression and therefore should be considered in the assessment of patient health status as well as in the design of treatment interventions and rehabilitation.

## 1. Introduction

Quality of life (QoL) is a useful endpoint in the study of multiple sclerosis (MS) not only as a prognostic factor but also as a quality marker of the health care provided. QoL in MS was found to be negatively affected by cognitive impairment [1] as well as by depression [2]. Depression is common in persons with MS, with a prevalence rate for major depression for MS patients in the community up to 25% [3]. Cognitive decline is also common in MS as prevalence rates in community samples of MS patients are reported around 40% [4, 5]. Cognitive impairment in MS patients could be measured with objective neuropsychological tests conducted by a clinical psychologist or measured by using self-answered questionnaires based on patients' subjective experience. Objective cognitive impairment was found to affect QoL in MS patient population [6], but subjective cognitive decline is not a well-established factor of decreased QoL in this patient group, despite the fact that it is easy to evaluate by using self-reported questionnaires. Subjective cognitive decline has also been suggested to be a consequence of depression [7, 8] and therefore should be taken into account in the treatment and rehabilitation processes. The aim of this study was to explore the hypothesis that subjective cognitive impairment affects QoL independently of depression.

## 2. Methods

### 2.1. Study Population

One hundred consecutive MS outpatients, 36 males and 64 females, took part in this study. All patients were diagnosed according to international diagnostic criteria [9]. Concurrent drug treatments with antidepressants, anticonvulsants, mood stabilizers, or disease-modifying therapies were permitted as patients were stabilized for at least one month in a stable treatment scheme. Patients taking benzodiazepines and/or antipsychotic agents were excluded from the study as these medications may affect cognitive function. The study protocol was approved by the ethical committee of our institution and informed consent was obtained from all participants.

### 2.2. Clinical Evaluation

The clinical evaluation included a formal neurological examination, QoL assessment, perceived cognitive impairment evaluation, and assessment of depression, as a part of a thorough medical examination which included assessment of disability by using the Expanded Disability Status Scale (EDSS). The self-reported questionnaires were filled by the MS patients, who received help from the researcher when necessary. The researchers were neurology registrars, who were under the supervision of a consultant neurologist. The assessments were performed in the outpatient clinic, in a quite office which was available for this study purposes.

### 2.3. Assessment of Disability

The Expanded Disability Status Scale (EDSS) [10] was used to assess and quantify the disability of MS patients. EDSS scores between 1.0 and 4.5 refer to MS patients who are fully ambulatory, whereas EDSS scores between 5.0 to 9.5 are defined by the impairment to ambulation. Current EDSS evaluation was made by a registrar neurologist during the same visit of the patient to the MS outpatient clinic.

### 2.4. Assessment of QoL

The patients were asked to complete the Short-Form-36 Health Survey (SF-36) [11]. SF-36 is a psychometric tool for the quantification of health status and quality of life (QoL) which consists of eight subscales: vitality, physical functioning, bodily pain, general health perceptions, physical role functioning, emotional role functioning, social role functioning, and mental health, each of them being the weighted sum of the questions on the corresponding section.

### 2.5. Assessment of Perceived Cognitive Decline

Patients were asked to complete the Perceived Deficits Questionnaire (PDQ) which is a self-report disease-specific questionnaire that measures the patients' perceived degree of cognitive impairment [12]. The subscales of the PDG, namely, PDQ-attention/concentration, PDQ-retrospective memory, PDQ-prospective memory, and PDQ-planning/organization correspond to the domains of perceived cognitive decline of the patients.

### 2.6. Assessment of Depression

Patients were asked to complete the depression subscale of the Mental Health Inventory (MHI), that is, a self-reported questionnaire for assessing the level of depression [13].

### 2.7. Statistical Analysis

After descriptive statistics and correlation analysis, univariate and multivariate linear regression was conducted to determine the best linear combination of age, gender, education, EDSS, depression, attention/concentration, retrospective memory, prospective memory, and planning/organization, for predicting quality of life scores in each SF-36 subscale. All statistical procedures were performed using the SPSS Statistics version 17.0 (SPSS Inc., Chicago, Ill.).

## 3. Results

### 3.1. Baseline Characteristics

Characteristics of the total sample of 64 female and 36 male patients with MS that fulfilled the inclusion criteria therefore included in the analysis are presented in Table 1. Of the initial number of 134 MS outpatients having been assessed as eligible for taking part in the study, no more than 9 refused to take part due to personal reasons or lack of time for answering the questionnaires, and no more than 25 were excluded by the researchers mainly due to heavy use of psychotropic medication affecting current mental state (e.g., benzodiazepines and/or antipsychotics). The between-gender comparison, using independent samples *t*-test, revealed no significant differences between gender subgroups. The Mann-Whitney test, a nonparametric equivalent of independent samples *t*-test, also showed no difference between genders, in the significance level of 0.05, in any of the variables presented in Table 1.

### 3.2. Correlation Analysis

**Figure 1:**
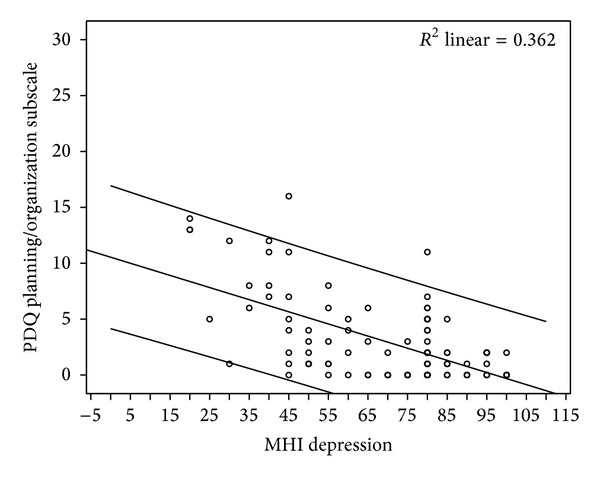
Scatter plot showing the relationship between depression and perceived impairment of planning/organization ability in population of patients with MS.

A correlation analysis using Pearson's *r* was conducted in which depression showed a low correlation with EDSS (*r* = 0.256, *P* < 0.05), but a moderate correlation with QoL (*r* = 0.502, *P* < 0.001), as well as with PDQ perceived impairment subscales: attention/concentration (*r* = 0.515, *P* < 0.001), retrospective memory (*r* = 0.445, *P* < 0.001), prospective memory (*r* = 0.483, *P* < 0.001), and planning/organization (0.601, *P* < 0.001).  Figure 1 shows the relationship between depression and perceived impairment of planning/organization ability in population of patients with MS. No significant correlations, in the level of 0.05, were found between depression and gender or age of the patients.

### 3.3. Regression Analysis

In Table 2 the results of the multiple linear regression analysis that was conducted in order to determine the best linear combination of age, gender, education, EDSS, depression, attention/concentration, retrospective memory, prospective memory, and planning/organization are presented, for predicting quality of life scores in all SF-36 subscales. This combination of variables predicts quality of life scores, with the variables significantly contributing to the prediction.

## 4. Discussion

Our data showed moderate correlations between depression and perceived cognitive impairment scales. Other studies also found a relationship between cognitive impairment and depression in MS population [7, 8, 14–22] but there is a need for cautious interpretation of such findings [7], as cross-section studies cannot determine direction of causality. Depression could lead to cognitive decline, a phenomenon also known as pseudodementia, but also impairment of cognition could be one of the first symptoms/criteria of depression. Nevertheless, some studies reported no significant relationship between cognitive impairment and depression [23, 24], by using different psychometric tools, populations, and study design, therefore not directly comparable to our data.

Our analysis showed that specific perceived cognitive impairments affect QoL independently of depression. Specifically, perceived impairment of planning/organization affects general health, vitality, social functioning, and role emotional QoL subscales independently of depression. In addition, perceived impairment of the retrospective memory affects general health and vitality subscales independently of depression. Interestingly, perceived attention/concentration dysfunction, as well as perceived prospective memory dysfunction, did not independently predict any QoL scores in our multinomial regression models. It seems that impairment in planning/organization and/or retrospective memory affects and decreases the QoL scales to a larger degree than do impairment of prospective memory and/or attention concentration.

Other studies also found self-reported cognitive impairment to correlate with impaired QoL [25], but a recent study found only weak associations between objective cognitive impairment and QoL [26]. These studies agree in the direction of the findings but differ in the degree of correlations, due to heterogeneity in the studied populations, in the fields of age, sex ratio, and the disease severity.

Depression is a well-known factor negatively affecting QoL and this is also confirmed in our data. We found evidence that depression predicts decreased QoL in the QoL scales of SF-36. Depression is a well-established QoL determinator not only in MS but also in many other medical conditions. Other studies have also demonstrated that depression affects QoL in MS population [6, 25, 27–29]. Furthermore a study reported no correlation between QoL scales and EDSS while controlling for depression and anxiety [30].

The strength of this study is that by involving a relatively large population of MS outpatients we explored associations between perceived cognitive impairment and QoL. To the knowledge of the authors this is the first evidence of this, and more extensive investigation is needed in order to delineate possible etiological factors as neurological and psychosocial correlates. A limitation of this study is that we did not include an additional objective neuropsychological assessment in order to explore the effects of objective cognitive decline on patients QoL as well as to compare the size of correlations between subjective and objective impairment in the explored areas of cognitive function. Even if this was not the point in this study, other authors already explored this area with contradictory results [20, 22, 25, 31–35], on whether MS patients are capable of self-estimating their objective cognitive decline.

Another limitation of this study is its cross-sectional design and the lack of second measurement, something that by definition yields bidirectional results. Cross-sectional design does not make it possible to explore for causal relationships between depression, cognitive impairment, and decline of QoL, despite the moderate correlation coefficients found. A prospective longitudinal design, using a structural equation model analysis, could probably define the unidirectional or bidirectional nature of these relationships.

Some authors suggested a common effect between depression and perceived cognitive impairment [31] but this is not extracted by the findings of this study as they remain independent predictors in the regression models. This finding suggests a separate role for perceived cognitive impairment that has to be further explored. Patient concerns related to the decline of their cognition should be interpreted with caution.

Perceived cognitive dysfunctions could be taken into account in the individualized rehabilitation plan in order to maximize rehabilitation's effectiveness on patients' QoL. During the rehabilitation process, clinicians should consider the possibility that cognitive testing confirms what the patients perceive regarding organizational skill deficits as well as the possibility that cognitive testing demonstrates that these cognitive abilities are preserved.

Perceived cognitive impairment in patients with MS constitutes a constellation of symptoms that the clinician should take into consideration. This could be a secondary sign of hidden depression but could also be an independent cognitive factor that is negatively affecting patients' quality of life. Both phenomena should be taken into account in the treatment process as well as in the design of rehabilitation programs.

## Highlights

- We examine effects of perceived cognitive impairment in QoL in a clinical sample of outpatients with MS.
- We also examine if these effects are independent of MS severity and depression.
- Perceived planning/organization dysfunction as well as perceived retrospective memory dysfunction independently predict QoL in MS.
- Perceived attention/concentration and prospective memory dysfunctions do not.
- Both of these perceived cognitive dysfunctions could be taken into account in the individualized rehabilitation plan in order to maximize rehabilitation's effectiveness on patients' QoL.

## Figures and Tables

**Table 1:** Characteristics of MS patients (*n* = 100).

Characteristics	Total sample	Males (*n* = 36)	Females (*n* = 64)	*P* value
Age, years	40.5 ± 10.3	40.9 ± 12.1	40.2 ± 9.2	NS
Education level (A/B/C) %	19/57/24	25/56/19	16/58/26	—
EDSS	3.6 ± 1.9	3.9 ± 2.2	3.4 ± 1.7	NS
MHI-depression	69.0 ± 21.0	71.7 ± 21.2	67.5 ± 20.9	NS
PDQ-attention/concentration	4.8 ± 4.0	4 ± 3.4	5.2 ± 4.3	NS
PDQ-retrospective memory	3.9 ± 4.3	3.2 ± 3.9	4.3 ± 4.5	NS
PDQ-prospective memory	2.8 ± 3.4	2.3 ± 2.9	3.1 ± 3.6	NS
PDQ-planning/organization	3.1 ± 3.8	2.4 ± 3.3	3.4 ± 4.0	NS
SF-36 physical functioning	66.0 ± 36.2	65.7 ± 42.1	66.1 ± 32.7	NS
SF-36 role physical	69.0 ± 42.4	72.2 ± 40.9	67.2 ± 43.4	NS
SF-36 general health	60.7 ± 20.1	63.9 ± 20.9	58.9 ± 19.6	NS
SF-36 vitality	55.0 ± 23.0	60.6 ± 23.8	51.9 ± 22.0	NS
SF-36 social functioning	72.4 ± 29.0	74.0 ± 30.1	71.5 ± 28.5	NS
SF-36 role emotional	75.0 ± 40.6	76.0 ± 40.3	74.5 ± 41.0	NS
SF-36 mental health	58.7 ± 17.5	62.9 ± 17.6	56.4 ± 17.2	NS

Unless specified otherwise, values are presented as means ± SD. Significance level or alpha (*α*) level was set at 0.05. Education level: A, primary education; B, secondary education; C, tertiary education; EDSS, Expanded Disability Status Scale; MHI, Mental Health Inventory; PDQ, Perceived Deficits Questionnaire, NS, nonsignificant.

**Table 2:** Simultaneous multiple regression analysis summary for age, education level, EDSS, depression, attention/concentration, retrospective memory, prospective memory, planning/organization, and predicting SF-36 QoL scales. Only significant factors appear in columns.

	MHI depression	PDQ-retrospective memory	PDQ-planning/organization
Physical functioning *R* ^2^ = 0.48, *F*_(9,86)_ = 10.90, *P* < 0.001			*B* = −3.29, SE = 1.57, beta = −0.35, *P* < 0.05
Role physical *R* ^2^ = 0.16, *F*_(9,86)_ = 3.04, *P* < 0.05	*B* = 11.29, SE = 4.87, beta = 0.28, *P* < 0.05		
General health *R* ^2^ = 0.32, *F*_(9,86)_ = 5.95, *P* < 0.001	*B* = 6.70, SE = 2.09, beta = 0.35, *P* < 0.05	*B* = 2.57, SE = 0.95, beta = 0.56, *P* < 0.05	*B* = −2.17, SE = 1.01, beta = −0.42, *P* < 0.05
Vitality *R* ^2^ = 0.47, *F*_(9,86)_ = 10.43, *P* < 0.001	*B* = 7.72, SE = 2.11, beta = 0.349, *P* < 0.001	*B* = 2.12, SE = .964, beta = 0.40, *P* < 0.05	*B* = −3.09, SE = 1.02, beta = −0.513, *P* < 0.05
Social functioning *R* ^2^ = 0.543, *F*_(9,86)_ = 13.53, *P* < 0.001	*B* = 11.05, SE = 2.48, beta = 0.40, *P* < 0.001		*B* = −3.83, SE = 1.19, beta = −0.51, *P* < 0.05
Role emotional *R* ^2^ = 0.347, *F*_(9,86)_ = 6.61, *P* < 0.001	*B* = 17.16, SE = 4.16, beta = 0.44, *P* < 0.001		*B* = −4.11, SE = 2.00, beta = −.0.38, *P* < 0.05
Mental health *R* ^2^ = 0.69, *F*_(9,86)_ = 23.99, *P* < 0.001	*B* = 12.81, SE = 1.24, beta = 0.77, *P* < 0.001		

EDSS, Expanded Disability Status Scale; MHI, Mental Health Inventory; SF-36, Short Form 36-item Health Survey; QoL, quality of life; PDQ, Perceived Deficits Questionnaire.
